# Language Dominance Modulates Transposed-Letter N400 Priming Effects in Bilinguals

**DOI:** 10.5334/joc.203

**Published:** 2022-01-07

**Authors:** Gabriela Meade, Jonathan Grainger, Phillip J. Holcomb

**Affiliations:** 1 Joint Doctoral Program in Language and Communicative Disorders, San Diego State University & University of California, San Diego, US; 2 Laboratoire de Psychologie Cognitive, CNRS & Aix-Marseille University, FR; 3 Department of Psychology, San Diego State University, US

**Keywords:** transposed-letter priming, orthographic precision, bilinguals, N400, ERPs

## Abstract

Models of visual word recognition differ as to how print exposure modulates orthographic precision. In some models, precision is the optimal end state of a lexical representation; the associations between letters and positions are initially approximate and become more precise as readers gain exposure to the word. In others, flexible orthographic coding that allows for rapid access to semantics (i.e., ‘good enough’ orthographic processing) is the optimal end state. To adjudicate between these trajectories, we compared the size of transposed-letter ERP priming effects on two ERP components thought to reflect orthographic and lexico-semantic processing across languages in late English-Spanish bilinguals. Words that are represented precisely should be less susceptible to activation by transposed-letter primes (e.g., *shpae-SHAPE*) than words that are not, and should therefore yield smaller priming effects. Overall, targets elicited smaller N250s and N400s and faster responses when preceded by transposed-letter primes compared to substitution primes (e.g., *shgue-SHAPE*). The only effect that significantly differed between languages was N400 priming, which was larger in English, the dominant language. We suggest that these results favor models of learning to read according to which ‘good enough’ orthographic processing increases with print exposure.

## Introduction

Bilingualism and second language learning are useful tools for investigating fundamental questions about language processing. In the present study, we compared word recognition in the first and second languages of late unbalanced English-Spanish bilinguals to determine how exposure to a written language changes orthographic processing. More specifically, we examined the degree of specificity with which letters are associated with positions within words, which we term orthographic precision. To index orthographic precision, we used a masked transposed-letter (TL) priming paradigm. TL primes formed by reversing two of the letters in a subsequent target word (e.g., *shpae-SHAPE)* facilitate target processing more than substitution primes in which those letters are replaced (e.g., *shgue-SHAPE*; Grainger et al., 2006; Ktori et al., 2014; Lupker et al., 2008; Meade et al., 2020; Meade et al., 2021; Perea & Carreiras, 2006, 2008; Perea & Lupker, 2004). This TL priming effect has been used to assert that orthographic processing does not involve precise one-to-one correspondences between letters and positions; if that were the case, the two types of primes would be equally similar to the target and should have equal influence on target processing. More recently, the relative size of TL effects across word forms and across individuals has been exploited as a measure of orthographic precision (e.g., Lally et al., 2020; Meade et al., 2020; Meade et al., 2021; Vergara-Martínez et al., 2013). The reasoning goes that words that are represented more precisely should be less robustly activated by TL primes and should therefore show smaller TL priming effects. Here, we apply this approach to an examination of the relationship between orthographic precision and exposure to a written language.

The question of how exposure to a written language modulates orthographic precision is an important one for dissociating among models of visual word recognition. Some models posit that increased exposure to words boosts precision, whereas others postulate that orthographic representations are more precise at early stages of learning. To illustrate, the lexical quality hypothesis is one model in which increased exposure leads to high-quality lexical representations that are precise and redundant (e.g., Perfetti, 1992, 2007). They are precise in that each letter is represented in its correct position and redundant in that the same fine-tuned form information is represented orthographically and phonologically. In contrast, in what we refer to as the optimal flexible coding hypothesis, the road to skilled reading involves learning to make use of *less* precise orthographic representations (Grainger et al., 2012; Grainger & Ziegler, 2011). That is, while beginning readers require precise information concerning letter positions to translate print to sound, more skilled readers can use approximate information about letter-in-word order as a fast track between print and meaning. Unique word identities can often be derived from a subset of the letters in the word together with information about their relative ordering and the overall word length, making these ‘good enough’ orthographic representations optimal for skilled silent reading. Such approximations can be achieved using open bigram coding (e.g., Grainger & van Heuven, 2004; Whitney, 2001), as in the dual-route orthographic coding model by Grainger and Ziegler (2011), but alternative mechanisms could also be applied without modifying the central hypothesis behind this account. In sum, the lexical quality hypothesis and the optimal flexible coding hypothesis assign divergent roles to precision within the orthographic system and make dissociable predictions about how exposure to a written language should affect this precision.

Several studies have addressed how precision changes as a function of print exposure by investigating how TL effects change over the course of reading development (Castles et al., 2007; Colombo et al., 2017; Comesaña et al., 2016; Lété & Fayol, 2013; Pagán et al., in press; Ziegler et al., 2014). For example, Ziegler and colleagues found that the size of behavioral TL priming effects increases from Grade 1 through Grade 5 (see also Colombo et al., 2017). In an ERP extension of this work, Eddy and colleagues (2016) found that the size of TL priming effects in children between the ages of eight and 10 were correlated with standardized behavioral measures of reading proficiency, such that stronger readers had larger effects.^1^ These changes are difficult to explain if we attribute TL priming to positional noise (e.g., Gómez et al., 2008), as that would imply that system noise increases over developmental time. However, the pattern falls out of the optimal flexible coding hypothesis: Children begin to maximize use of flexible, ‘good enough’ orthographic representations as they become mature readers (e.g., Grainger & Ziegler, 2011), which in turn increases the effectiveness of TL primes and the relative size of TL priming effects (Colombo et al., 2017; Eddy et al., 2016; Ziegler et al., 2014). This transition might well occur at different time points and to different degrees for individual words; recent studies have demonstrated that TL priming can be used to index variations in orthographic precision as a function of lexical-level characteristics, even in adult readers (e.g., Lally et al., 2020; Meade et al., 2021; Vergara-Martínez et al., 2013).

Extending this literature then, the goal of the present study was to investigate how orthographic precision in adults is modulated by print exposure. To this end, we compared the size of TL priming effects for native language (L1) and second language (L2) words in late unbalanced English-Spanish bilinguals who performed a language decision task. We measured TL priming in terms of behavioral facilitation and mean amplitude within the N250 and N400 windows of the ERP waveform. Generally, targets preceded by TL primes elicit smaller amplitude negativities than those preceded by substitution primes within one or both of these time windows (e.g., Carreiras, Duñabeitia, et al., 2009; Carreiras, Vergara, et al., 2009; Grainger et al., 2006; Ktori et al., 2014; Meade et al., 2020; Meade et al., 2021; Vergara-Martínez et al., 2013). The smaller amplitude negativities are interpreted in terms of facilitated processing due to pre-activation of target representations by the primes. Whereas the N250 effect is associated with the transition from sublexical to lexical processing, the N400 effect is associated with later lexico-semantic processing (see, e.g., Grainger & Holcomb, 2009). Since the lexical quality hypothesis posits that precision is the optimal end state of lexical representations, the orthographic precision for a given word should increase as the reader gains exposure to it. Thus, this model postulates that written words in the L1 will have *more* precise representations overall that generate *smaller* TL priming effects than in the L2. Moreover, within the L2, participants who are more proficient might show smaller TL priming effects than those who are less proficient. In contrast, the optimal flexible coding hypothesis posits that ‘good enough’ orthographic codes provide an optimal means to access to semantic representations from print when reading familiar words. Following this logic, written words in the L1 would be expected to make greater use of this more efficient ‘good enough’ orthographic processing, yielding *less* precise representations that generate *larger* TL priming effects than in the L2. Within the L2, participants who are more proficient would also be expected to have larger TL priming effects than those who are less proficient. Thus, examining the influence of exposure on the size of the priming effect within and across languages can help us infer what underlying mechanisms are at play.

## Methods

### Participants

Data were analyzed from 20 late English-Spanish bilinguals (14 female; mean age 24.5 years, *SD* 4.3 years) in the San Diego area who considered themselves to be proficient in L2 Spanish. All participants had been exposed to English since birth. In contrast, they began learning Spanish at age 11 or later (average age of Spanish acquisition: 14.3 years, *SD* 3.0 years). As presented in ***Table 1***, self-reported proficiency data for both languages confirmed that participants perceived themselves as more proficient in L1 English than in L2 Spanish at the time of testing. On a dominance scale from 1 (Spanish) to 9 (English), the mean rating was 7.6 (*SD* 0.9). As an objective measure of proficiency, the LexTALE was administered in both languages (see ***Table 1***; Izura et al., 2014; Lemhöfer & Broersma, 2012).^2^ All participants were right-handed and had normal or corrected-to-normal vision. An additional five participants who met these criteria were excluded for low accuracy on the translation post-test (<50%; see Procedure below) and two were excluded due to high artifact rejection rates in the ERP task.

**Table 1:** Mean (SD) self-reported proficiency (1 = unable, 5 = expert) and LexTALE scores in L1 English and L2 Spanish.


	READING	SPELLING	SPEAKING	LISTENING	LEXTALE

**L1 English**	5.00 (0.00)	4.55 (0.83)	4.90 (0.31)	4.95 (0.22)	93.94 (7.02)

**L2 Spanish**	3.80 (0.77)	4.05 (1.15)	3.80 (0.77)	3.70 (0.66)	65.92 (7.58)


### Stimuli

Targets were 50 L1 English and 50 L2 Spanish words that were five letters long and had a noun meaning. Using the respective SUBTLEX databases, frequency in occurrences per million was controlled between the L1 English and L2 Spanish targets, *t*(98) = .03, *p* = .976 (see ***Table 2***; Brysbaert & New, 2009; Cuetos et al., 2011). That said, presumably the L2 Spanish words had a lower subjective frequency for our participants given their language dominance profile. In light of recent evidence that TL priming effects differ as a function of orthographic neighborhood density (Meade et al., 2021), OLD20 was also balanced between L1 English and L2 Spanish targets, *t*(98) = .09, *p* = .930. OLD20 is a measure of orthographic neighborhood density that is calculated by taking the average Levenshtein distance (i.e., number of additions, deletions, substitutions) between the target and the 20 words that are most similar to it in the lexicon. Given the bilingual status of our participants, we calculated OLD20 based on a lexicon that included all words between three and eight letters long in the respective databases for the two target languages (Balota et al., 2007; Duchon et al., 2013). Language-specific OLD20s for the two sets of targets are also reported for reference in ***Table 2***.

**Table 2:** Characteristics of the L1 English and L2 Spanish targets and primes [mean (SD)].


TARGET	OLD20	OLD20(L1 LEXICON)	OLD20(L2 LEXICON)	FREQUENCY	TL PRIME OLD20	SUB PRIME OLD20

L1 English	1.66 (.17)	1.74 (.14)	2.08 (.29)	64.11 (113.63)	1.98 (.18)	2.03 (.18)

L2 Spanish	1.66 (.17)	2.14 (.35)	1.73 (.14)	64.86 (132.67)	1.95 (.16)	2.01 (.20)


**Note**: Unless otherwise specified, OLD20 was calculated from a lexicon that includes both L1 English and L2 Spanish words to best approximate the bilingual lexicon of our participants.

Each of the 100 targets was preceded by a TL prime and a substitution prime for a total of 200 trials. TL primes were formed by reversing two word-internal letters; half of the transpositions in each language were between the 2^nd^ and 3^rd^ positions and half of them were between the 3^rd^ and 4^th^ positions. The transpositions were always between a consonant and a vowel. Substitution primes were formed by replacing the letters in the same position such that the consonant/vowel structure and visual outline (i.e., ascenders and descenders) were identical to the TL primes. The positions and letters that were transposed were identical across trials in the two languages to minimize potentially confounding effects of sublexical regularity. None of the primes were words in either language and the orthographic neighborhood density of the two types of primes that occurred before targets in each language was controlled (see ***Table 2***), both *p*s > .10.

### Procedure

The trial structure was identical to our previous masked TL priming study (Meade et al., 2021). Each trial began with a white fixation cross presented at the center of the screen for 500 ms. A forward mask (#######) then appeared for 300 ms, followed by a lowercase prime for 50 ms, a backward mask (#######) for 20 ms, and an uppercase target for 300 ms. After a response was registered, the screen remained blank for 750 ms and then a purple fixation cross appeared at the center of the screen for 1500 ms. Participants were instructed to blink during this purple cross in between trials and during occasional longer breaks. Stimuli were displayed in Courier font such that they subtended a horizontal visual angle of 1.7 degrees.

On each trial, participants performed a language decision task on the targets (i.e., no mention was made of the prime). They pressed one button on a videogame controller for Spanish words and another button for English words, with response hand counterbalanced across participants. Trials were presented in two possible pseudorandomized list orders such that targets were presented with a substitution prime in one half of the list and with a TL prime in the other half. The prime with which each target first occurred was counterbalanced across lists. Orthographic and semantic relatedness of the targets was minimized between consecutive trials and no more than three consecutive trials belonged to the same language. The experiment began with a practice list containing 10 trials, half of which had L2 Spanish targets.

Critically, this language decision task could have been done with no knowledge of Spanish (i.e., press one button for familiar L1 English words and the other button for unfamiliar words). To confirm that participants were indeed familiar with the L2 Spanish targets, they were asked to translate them into L1 English immediately following the ERP task. On average, participants provided a translation for 38.3 (*SD* .76) of the 50 Spanish words. This relatively low accuracy is the tradeoff of recruiting late bilinguals who were strongly L1 English dominant. Trials with unfamiliar L2 Spanish words (i.e., that were not translated) as targets were excluded from the analyses reported below.

### EEG Recording and Analysis

Participants wore an elastic cap (Electro-Cap) with 29 electrodes and four additional electrodes. The electrode on the left mastoid was used as a reference during recording and for all subsequent analyses. The electrode on the right mastoid was used to monitor differential mastoid activity, which was not observed. The electrode below the left eye was used to identify blinks in conjunction with the recordings from FP1 and the electrode next to the outer canthus of the right eye was used to monitor horizontal eye movement. Using saline gel (Electro-Gel), the impedances of all electrodes were maintained below 2.5 kΩ. EEG was amplified with SynAmpsRT amplifiers (Neuroscan-Compumedics) with a bandpass of DC to 100 Hz and was sampled continuously at 500 Hz.

**Figure 1:**
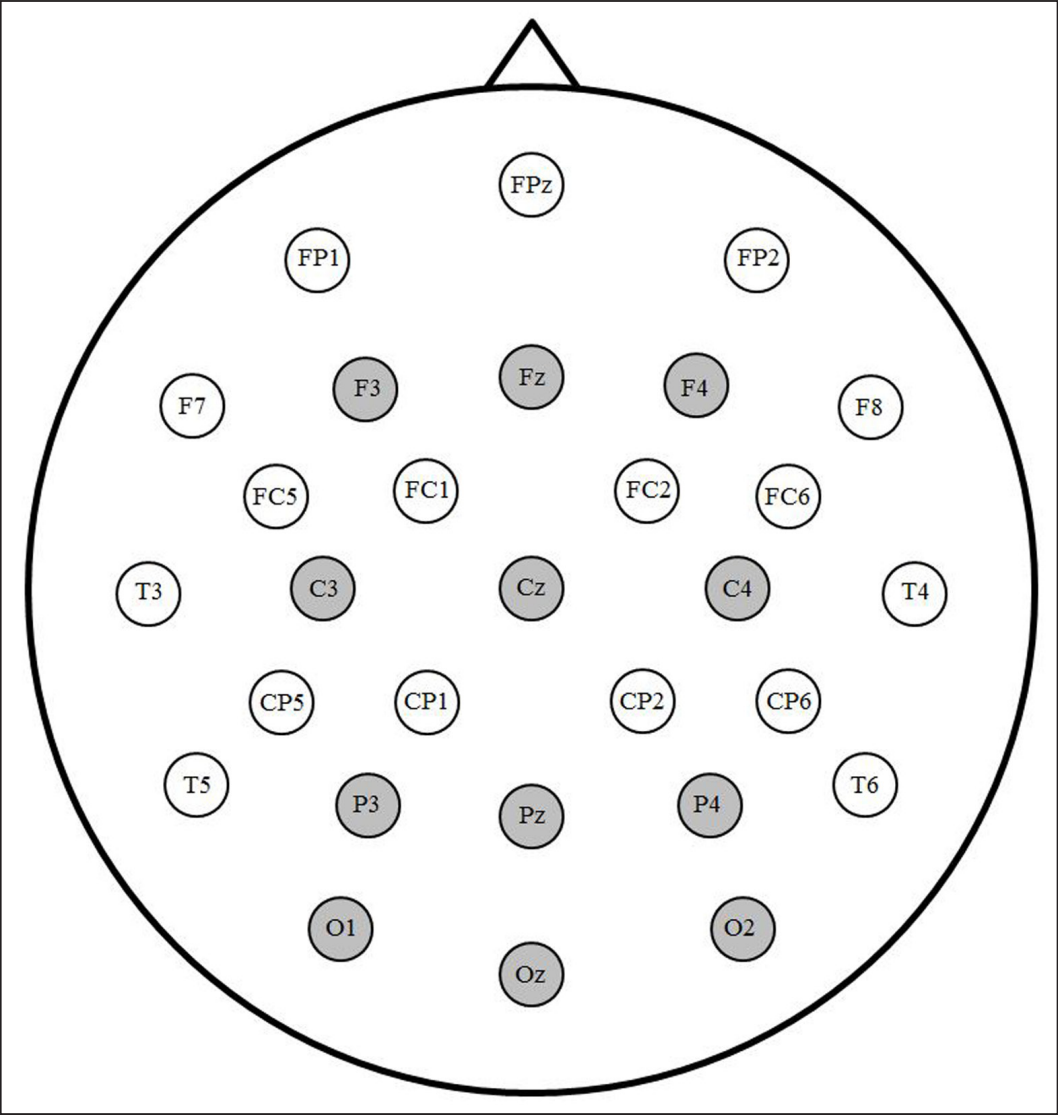
Electrode montage. Sites highlighted in gray were included in analyses.

ERPs were time-locked to target onset and low-pass filtered at 15 Hz. Epochs spanned 1000 ms including a 100 ms pre-target-onset baseline. Trials with artifacts related to eye movement or drift during this epoch (9.5 trials, or 5.3%, on average) were excluded from analyses, as were trials with incorrect language decision responses. Separate ERPs for each language and prime combination were averaged for each participant and each of the 12 representative electrodes depicted in ***Figure 1***. Mean N250 amplitude was calculated between 150 and 275 ms and mean N400 amplitude was calculated between 350 and 550 ms, consistent with our previous masked TL priming study (e.g., Meade et al., 2021). For each time window, repeated-measures ANOVAs were conducted with within-subject factors Language (L1, L2), Prime (TL, Substitution), Laterality (Left, Midline, Right) and Anterior/Posterior (Frontal, Central, Parietal, Occipital). Greenhouse-Geisser correction was applied for all within-subject measures with more than one numerator degrees of freedom. Partial eta squared (η_p_^2^) is reported as a measure of effect size.

## Results

### N250 Amplitude

**Figure 2:**
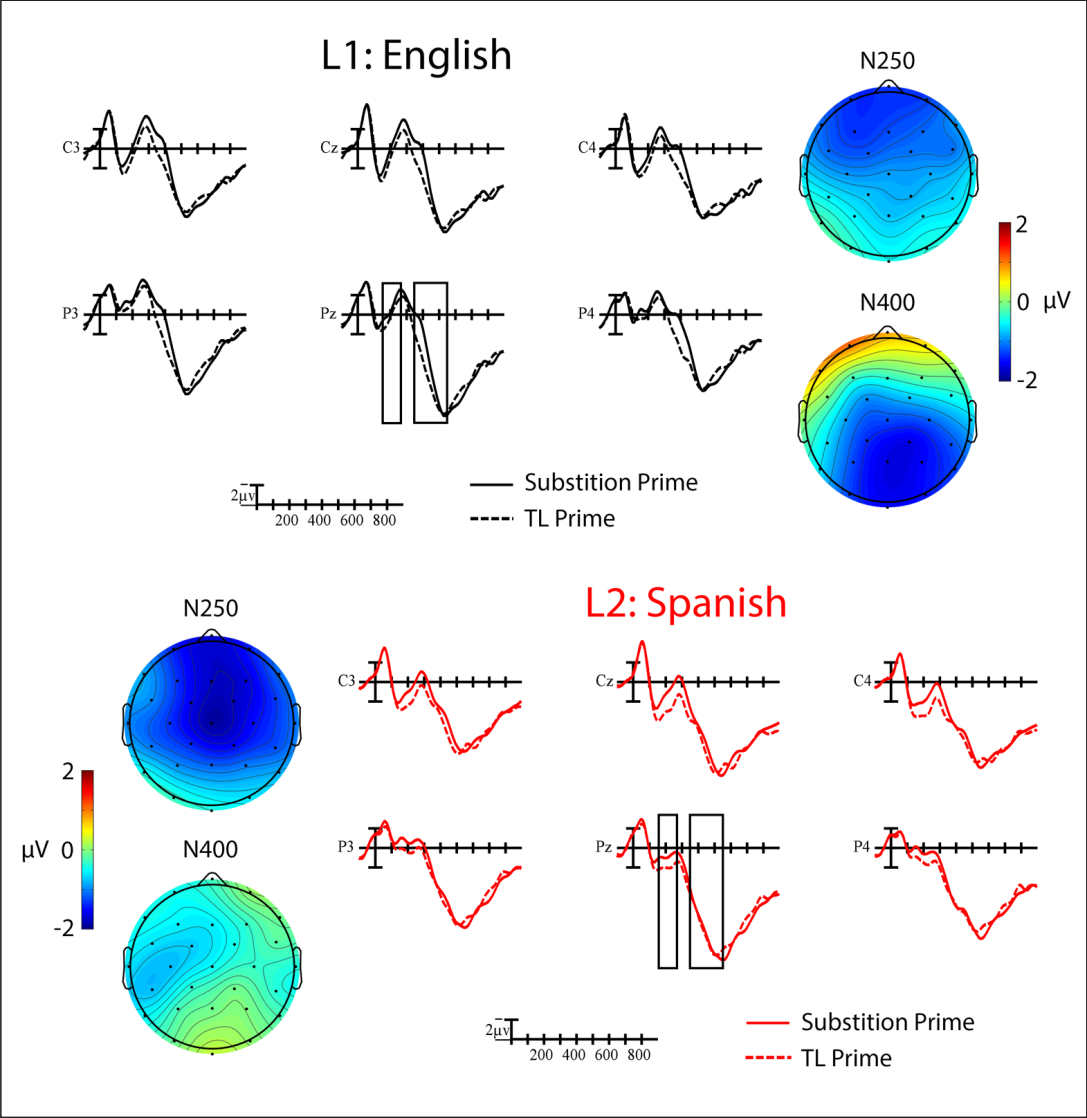
Grand average ERP waveforms showing the main effect of TL priming for word targets in L1 English (black) and L2 Spanish (red). Targets preceded by TL primes (dotted lines) elicited smaller amplitude negativities than those preceded by substitution lines (solid lines). Each vertical tick marks 100 ms and negative is plotted up. The vertical line marks target onset and the calibration bar marks 2 µV. The N250 and N400 windows that were analyzed are indicated with the black boxes. The scalp voltage maps show the distribution of the priming effects (substitution-TL) in each time window for the two languages.

**Figure 3:**
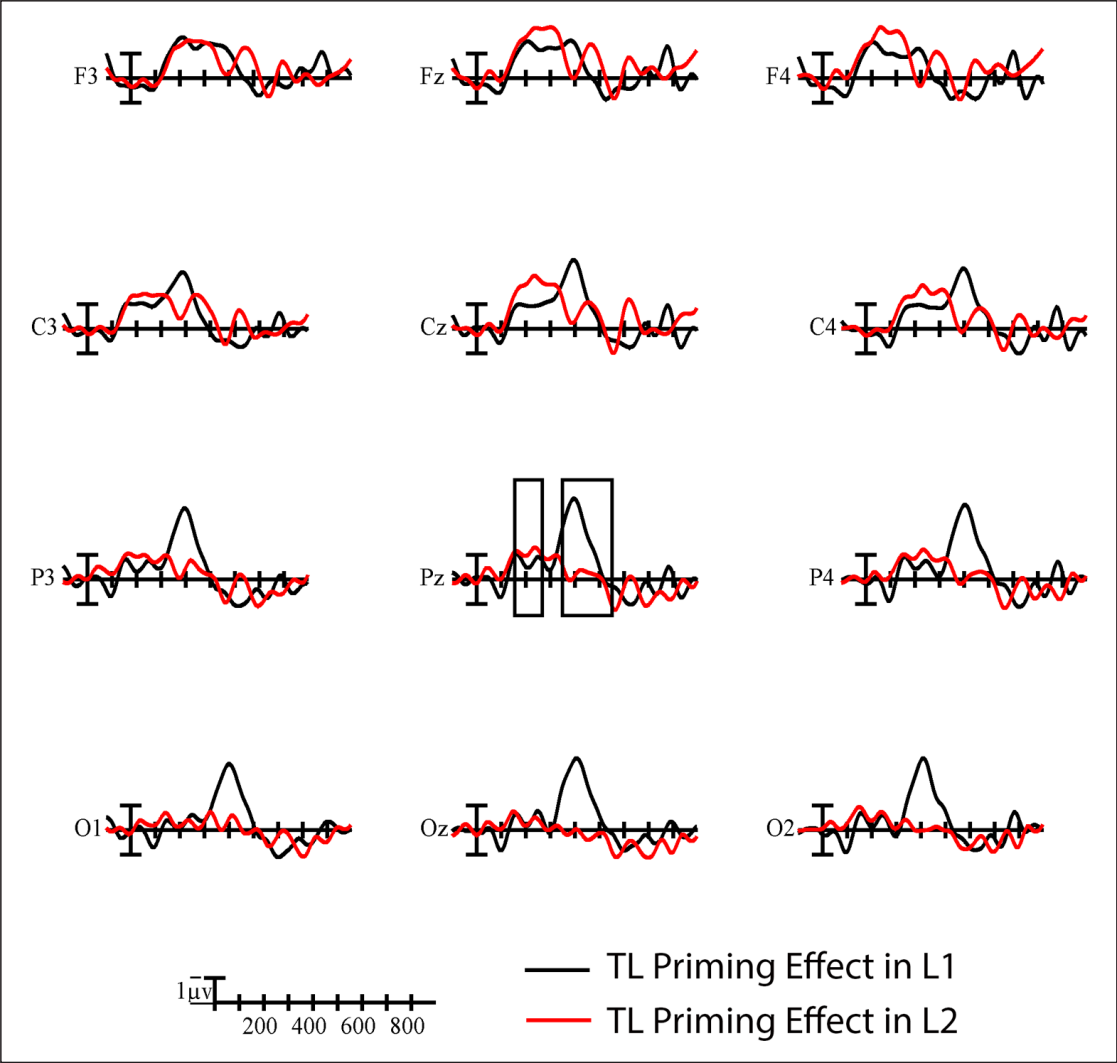
Difference waves showing the time course of TL priming effects (substitution-TL) in L1 English (black) and L2 Spanish (red) at all analyzed electrode sites. Each vertical tick marks 100 ms and negative is plotted up. The calibration bar marks 1 µV. The N250 and N400 windows are indicated by the black boxes at representative site Pz.

**Figure 4:**
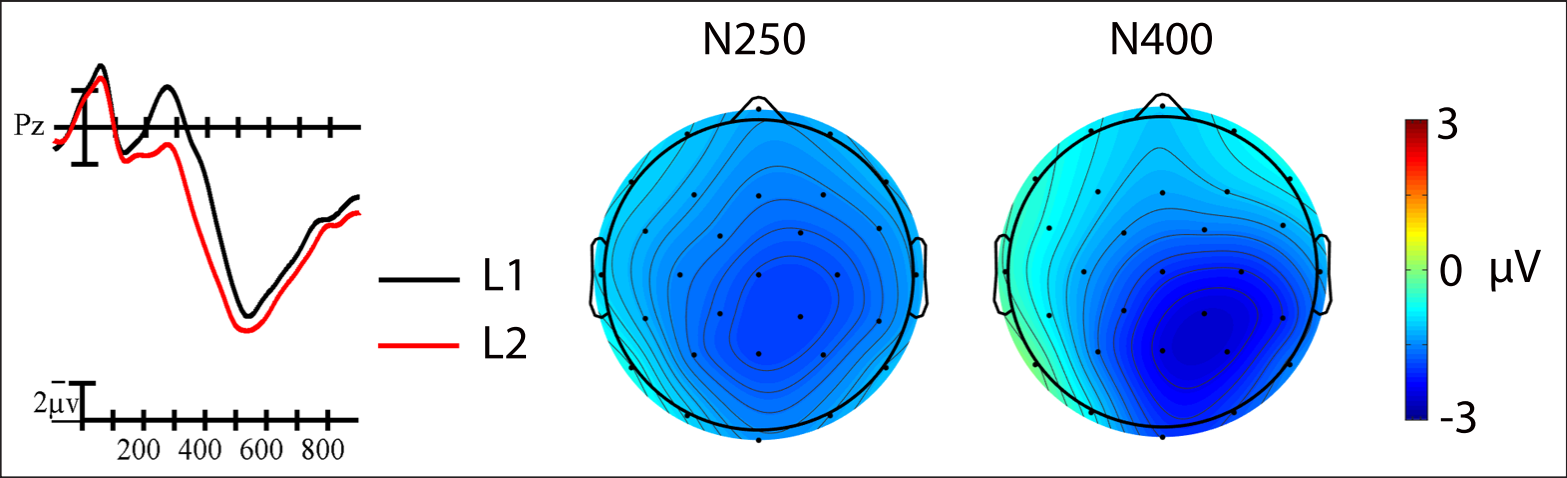
The main effect of Language at representative site Pz. L1 targets (black line) elicited larger amplitude negativities than L2 targets (red line) overall. Each vertical tick marks 100 ms and negative is plotted up. The vertical line marks target onset and the calibration bar marks 2 µV. The scalp voltage maps show the distribution of the effects (L1-L2) within the N250 and N400 windows that were analyzed.

A significant main effect of Prime indicated that targets preceded by TL primes elicited smaller amplitude N250s than those preceded by substitution primes overall, *F*(1,19) = 7.92, *p* = .011, η_p_^2^ = .29 (see ***Figures 2*** and ***3***). The TL priming effect was largest across anterior sites, Prime × Anterior/Posterior, *F*(3,57) = 8.92, *p* = .002, η_p_^2^ = .32. A significant main effect of Language indicated that L1 English targets elicited larger amplitude negativities within the N250 window than L2 Spanish targets, *F*(1,19) = 13.50, *p* = .002, η_p_^2^ = .42 (see ***Figure 4***). Critically, none of the interactions between Language and Prime were significant, all *p*s > .09.

### N400 Amplitude

A significant main effect of Prime indicated that targets preceded by TL primes elicited smaller N400s than those preceded by substitution primes, *F*(1,19) = 4.97, *p* = .038, η_p_^2^ = .21 (see ***Figure 2***). The distribution of the TL priming effect differed across languages, Language × Prime × Anterior/Posterior, *F*(3,57) = 5.20, *p* = .020, η_p_^2^ = .22. To better characterize this interaction, we conducted follow-up analyses at each level of the Anterior/Posterior factor. The Language × Prime interaction was only significant at occipital sites, where the TL priming effect was larger in English than in Spanish, *F*(1,19) = 6.09, *p* = .023, η_p_^2^ = .24 (see ***Figure 3***). Using the Spanish LexTALE as an objective measure of L2 proficiency, we calculated the correlation between proficiency and the mean size of the L2 priming effect (substitution – TL) at occipital sites. There was a moderate negative correlation between these values that did not reach significance, *r* = –.29, *p* = .22. This suggests that the participants with more proficiency (i.e., higher LexTALE scores) tended to have larger priming effects in the expected direction (i.e., a larger negative difference between conditions). Finally, a significant main effect of Language indicated that L1 English targets continued to elicit larger negativities than L2 Spanish targets into the N400 window, *F*(1,19) = 8.97, *p* = .007, η_p_^2^ = .32 (see ***Figure 4***). This difference was especially large across parietal sites, Language × Anterior/Posterior, *F*(3,57) = 3.80, *p* = .045, η_p_^2^ = .17.

### Behavior

Mean reaction time and accuracy in the language decision task for each condition are reported in ***Table 3***. There was a significant main effect of Prime such that targets preceded by TL primes elicited faster responses than targets preceded by substitution primes overall, *F*(1,19) = 4.57, *p* = .046, η_p_^2^ = .19. Although the size of the effect was numerically larger in English (16 ms) than in Spanish (4 ms), it did not significantly differ between the two languages, *F*(1,19) = 2.21, *p* = .154, η_p_^2^ = .14. The main effect of Language on RTs also failed to reach significance, *F*(1,19) = 1.36, *p* = .26, η_p_^2^ =.06. Neither of the factors had a significant effect on accuracy in the language decision task, all *p*s > .13.

**Table 3:** Behavioral results [mean (SD)].


TARGET	PRIME	RT (MS)	ACCURACY (%)

L1 English	TL	602 (72)	96.8 (3.7)

	Substitution	618 (84)	95.7 (4.2)

L2 Spanish	TL	596 (67)	96.9 (3.2)

	Substitution	600 (62)	95.9 (3.2)


## Discussion

Comparing TL priming effects between languages in bilinguals enabled us to investigate how orthographic precision changes as a function of exposure to printed words. We found significant priming across the N250 and N400 windows and reaction times, but the size of the priming effect only significantly differed between languages in the N400 window (see ***Figure 3***). The direction of this interaction is key for determining how exposure to a written language modulates orthographic precision. In line with the optimal flexible coding hypothesis, the comparatively larger N400 TL priming effects in the L1 indicate that increased exposure to a written word increases the flexibility with which it is represented. The ‘good enough’ orthographic codes that provide a fast track to semantics have been optimized for the L1 words. In contrast, reduced exposure to the L2 in our late unbalanced bilinguals gave them less opportunity to optimize word recognition in this way. In the context of this experimental paradigm, the flexible codes that predominate L1 processing can more readily be activated by the TL primes, yielding larger TL priming effects in that language. Although the correlation between proficiency and the size of the L2 priming effect did not reach significance, the overall pattern within the L2 supports similar reasoning. The participants who scored higher on the L2 LexTALE and benefited from the opportunity to maximize word processing efficiency in the L2 tended to have a larger L2 priming effect than those who had lower LexTALE scores.

The dual-route model proposed by Grainger and Ziegler (2011) formalized the idea that visual word recognition can proceed via sublexical orthographic representations that differ in precision. In the original instantiation of the model, use of the two routes was jointly determined by reading skill and the task at hand. The sublexical orthography-to-phonology links along the fine-grained route were hypothesized to require precise information about the position of each letter (particularly important for processing multi-letter graphemes) and to be critical for beginning readers and reading aloud. In contrast, the ‘good enough’ orthographic representations along the coarse-grained route were hypothesized to provide more efficient, direct access to semantics during skilled silent reading. In a recent update to this model, we proposed that lexical characteristics (e.g., neighborhood density) determine the relative connection strengths between precise versus ‘good enough’ sublexical orthographic representations and the corresponding whole-word orthographic representations (Meade et al., 2021). That is, the two routes of the original model are replaced by variations in connection strengths between sublexical orthographic representations (that vary in positional precision) and whole-word orthographic representations. Becoming a skilled reader involves maximizing the efficiency with which meaning can be accessed by transitioning to greater use of more flexible sublexical orthographic representations where appropriate. The key role played by connection strengths between sublexical and lexical representations proposed by Meade et al. (2021) is particularly important for interpreting the results of the present study. It is the word-specific (and therefore, by extension, language-specific) connectivity between sublexical orthographic representations and whole-word representations that explains why, in the same participants, TL effects were found to differ across languages.

Based on the electrophysiological timeline of visual word recognition introduced by Grainger and Holcomb (2009), we originally expected this interactivity to arise within both the N250 and the N400 windows. Recall that these are the two ERP components that they associated with interactive processing between sublexical orthographic representations and whole-word orthographic representations. Whether or not an effect is seen on either one or both of these components might depend on the relative impact of purely sublexical and purely lexical processing on the effect under investigation and the amount of feedforward versus feedback processing that is involved. The N250 would be mostly sensitive to purely sublexical processing as well as feedback from lexical to sublexical representations, while the N400 would be mostly sensitive to lexico-semantic processing and fast feedforward activation of lexical representations. To some extent, the balance of these factors – and therefore the temporal manifestation of the interaction of interest – must also depend on the task demands and the particular population being investigated. Thus, in an ERP investigation of beginning readers (Eddy et al., 2016), the impact of reading fluency on the size of TL effects was seen in both the N250 and N400 components. In an investigation of the modulation of TL priming effects by orthographic neighborhood density (Meade et al., 2021), the predicted interaction was only found in the N250 time window. Apart from the different populations being investigated, one key difference between the Meade et al. study and the present investigation is the task that participants had to perform – lexical decision in Meade et al. and language decision in the current study. Different strategies across these tasks could well influence the depth with which the stimuli are processed and therefore impact the precise timing of the observed pattern of ERP effects.

Not finding the critical interaction between TL priming and language within the N250 window rules out an interpretation that relies solely on sublexical dynamics. Both languages use the same alphabet, but previous research indicates that language-specific bigram frequencies can influence processing in bilinguals (see also Casaponsa et al., 2014; Oganian et al., 2016; van Kesteren et al., 2012). Increased exposure to L1 words necessarily entails increased exposure to sublexical patterns that predominantly occur in the L1, which theoretically could have influenced differences in processing between languages. In a post-hoc analysis based on the three bigram measures in the English Lexicon Project (i.e., sum and mean of the bigram count and sum of the bigram count by position; Balota et al., 2007), we confirmed that the targets in the two languages were similarly “English like” at a sublexical level, all *p*s > 0.49. Interestingly, the same did not hold true in Spanish. Using the mean type and token bigram counts by position available in BuscaPalabras (Davis & Perea, 2005), the Spanish targets were significantly more “Spanish like” than the English targets, *p*s < .001. Thus, an effect of sublexical orthographic markedness cannot be ruled out and could in fact explain why we found numerically faster language decision latencies for Spanish (L2) words relative to English (L1) words. However, we fail to see how this could be driving the significantly greater N400 TL priming effects in L1 compared to L2. Instead, we would argue that our results point to the mapping of sublexical orthographic representations onto whole-word orthographic representations, and the relative strengths of the connections involved in this mapping, as the explanation for how TL priming effects are modulated by reading experience. With more reading experience, the connections between flexible sublexical orthographic representations (e.g., open bigrams) and whole-word orthographic representations are strengthened, hence leading to greater TL priming effects in the L1, and particularly in the N400 time window.

In the present study, we used language in late unbalanced bilinguals as a proxy for exposure to the written words that were included in the experiment. The finding of a significant interaction within the N400 window prompts the question of whether or not a similar relationship between exposure and orthographic precision exists *within* a language. As reviewed in the Introduction, older and more skilled developing readers appear to use more flexible orthographic codes, as reflected in the size of TL priming effects (e.g., Colombo et al., 2017; Eddy et al., 2016; Ziegler et al., 2014). In adult readers, this might be expected to translate to more flexible orthographic codes for high frequency words relative to low frequency words.^3^ Behavioral evidence for an interaction between frequency and the size of TL priming effects is elusive (e.g., Forster et al., 1987; Yang & Lupker, 2020). Note, however, that the interaction between language and TL priming also failed to reach significance in the language decision responses that we analyzed here. In contrast, in the only ERP study to address this issue, Vergara-Martínez and colleagues (2013) reported that N400 amplitude and reaction times differentiated TL nonwords (e.g., BRIGDE) from substitution nonwords (e.g., BRITGE) when the base words had a high frequency, but not when they had a low frequency. In other words, increased exposure to the high frequency words appears to generate more flexible representations that were more susceptible to be activated by the TL nonword, in line with the optimal flexible coding hypothesis. That is, high frequency words would provide readers with more opportunities for reinforcing the use made of flexible, ‘good enough’ orthographic representations.

Alternatively, one could argue that the larger TL effects for high frequency words in the study by Vergara-Martínez et al. (2013), and for the L1 words in the present study, were simply caused by faster lexical access. That is, increased exposure in the L1 would lead to greater connection strengths between sublexical and lexical representations, which would allow TL nonwords to better activate the corresponding base words. Therefore, stronger connection strengths in L1 compared with L2, combined with a model of noisy letter position coding such as the Overlap model (Gómez et al., 2008), could well account for the present findings. Under this alternative account and our own account, changes in the size of TL effects reflect some form of orthographic learning that results in changes in the connection strengths between sublexical orthographic representations and whole word orthographic representations. The key difference between the two accounts is that according to the ‘speed of lexical access’ account the changes in connection strengths would operate across the board (i.e., everything gets faster in L1), whereas according to our optimal flexible coding hypothesis these changes operate at the individual word level. Combining the optimal flexible coding hypothesis with an integrated lexicon account of bilingual lexical representation (Jacobs & Grainger, 1992; van Heuven et al., 1998) leads to the interesting prediction that cross-language neighborhood density should impact on TL effects in bilingual people, in the same manner as found in monolingual participants when applying a within-language manipulation of neighborhood density (Meade et al., 2021).

Taken together, the results presented here support the optimal flexible coding hypothesis, in which the increased exposure associated with the more proficient L1 is postulated to generate decreased orthographic precision. Connection weights between flexible sublexical representations and whole word representations become strengthened on a word-by-word basis, subsequently generating the differences that we detected between languages. To a lesser degree, there was evidence of proficiency within the L2 modulating orthographic processing in a similar manner. More generally, these results provide an example of how the unique linguistic experiences of bilinguals can be used to address fundamental questions about visual word processing.

## Data Accessibility Statement

The stimuli and data relating to this project can be found at *https://osf.io/pn5yf/*.

## Footnotes

We acknowledge that TL effects obtained to nonword stimuli in unprimed lexical decisions have been found to diminish with an increase in one measure of reading skill in Grade 6 children (Gómez et al., 2021). However, the fact that the measure in question was pseudoword reading ability actually fits well with the Grainger and Ziegler (2011) framework, since pseudoword reading should require more precise orthographic representations.

The formulas recommended to score the two versions of the LexTALE are different. To calculate scores that were more obviously comparable across the two tests, which have different numbers of items, we summed across the percentage of correct words and nonwords. A perfect score was therefore 100 across the two tests.

Despite matching frequency between the two languages, subjective frequency of the individual words (i.e., comparatively less exposure to the L2 Spanish words in these late unbalanced bilinguals) may have influenced the patterns that we report here. However, it seems unlikely that our results are solely driven by frequency. If that were the case, then we would have expected the lower frequency (i.e., L2) words to elicit larger amplitude N400s (e.g., Dufau et al., 2015; Rugg, 1990). Rather, we replicated the standard pattern in late bilinguals of a smaller amplitude N400 for L2 words relative to L1 words (e.g., Midgley et al., 2009; Soskey et al., 2016).
